# Bidirectional propagation of low frequency oscillations over the human hippocampal surface

**DOI:** 10.1038/s41467-021-22850-5

**Published:** 2021-05-12

**Authors:** Jonathan K. Kleen, Jason E. Chung, Kristin K. Sellers, Jenny Zhou, Michael Triplett, Kye Lee, Angela Tooker, Razi Haque, Edward F. Chang

**Affiliations:** 1 Department of Neurology, Weill Institute for Neurosciences, University of California San Francisco, San Francisco, CA USA; 2 Department of Neurological Surgery, Weill Institute for Neurosciences, University of California San Francisco, San Francisco, CA USA; 3 Lawrence Livermore National Laboratories, Livermore, CA USA

**Keywords:** Computational neuroscience, Hippocampus

## Abstract

The hippocampus is diversely interconnected with other brain systems along its axis. Cycles of theta-frequency activity are believed to propagate from the septal to temporal pole, yet it is unclear how this one-way route supports the flexible cognitive capacities of this structure. We leveraged novel thin-film microgrid arrays conformed to the human hippocampal surface to track neural activity two-dimensionally in vivo. All oscillation frequencies identified between 1–15 Hz propagated across the tissue. Moreover, they dynamically shifted between two roughly opposite directions oblique to the long axis. This predominant propagation axis was mirrored across participants, hemispheres, and consciousness states. Directionality was modulated in a participant who performed a behavioral task, and it could be predicted by wave amplitude topography over the hippocampal surface. Our results show that propagation directions may thus represent distinct meso-scale network computations, operating along versatile spatiotemporal processing routes across the hippocampal body.

## Abstract (web summary)

New microgrid recordings on the human hippocampal surface reveal that oscillations travel in reversing directions. The route of travel at a given moment was related to behavior and topographic patterns of activity strength, suggesting directions may be biomarkers of hippocampal cognitive processes.

## Introduction

The hippocampus has a diverse connectivity landscape with cortical and subcortical brain regions along its longitudinal axis^1–4^. This gives rise to a functional continuum between the temporal and septal poles; for example, stronger representations of emotional and visuospatial processing, respectively^5–8^. While the hippocampus thereby appears to have a modular substructure serving separate brain systems, it also provides an interface along its axis for integrative processing^4,5,9,10^. For example, the encoding and other processing of an episodic memory (e.g., losing one’s cell phone in Times Square) might be dominated by either the subjective distress of the experience (emotional information) or the objective place or context (visuospatial information) but, most crucially, a fusion of both is achieved.

How such multimodal integration might be coordinated across the hippocampal body is unclear, however. Traveling waves (TWs) are one potential mechanism, manifested as singular waves or repeated cycles of voltage changes propagating across tissue, sequentially affecting the timing of neuronal activity^11–16^. Multi-site hippocampal depth electrode recordings in rodents and humans have indeed reported theta oscillations (4–10 Hz) propagating from the septal pole to the temporal pole^12,15,16^. This spatiotemporal organization may be fundamental for cognitive integration and phase-dependent neuronal information encoding^5,11,15^.

Yet, other reports in rats suggest that intrinsic hippocampal neural activity may not flow in only one direction^17,18^. Recent methods applying two-dimensional models to electrode grid recordings over the cortical surface have revealed oscillations traveling in *multiple* directions supporting versatile inter-regional communication and timing^13,19–23^. We hypothesized that analogous grid recordings over the extensive human hippocampal surface^24,25^ may reveal other versatile wave properties relevant for the flexible assimilation of its topographical connections. We also sought to understand whether wave propagation extends to low frequencies in delta (1–4 Hz) and alpha (10–15 Hz) ranges, since they may herald distinct cortico-hippocampal communications^26–28^.

**Fig. 1:**
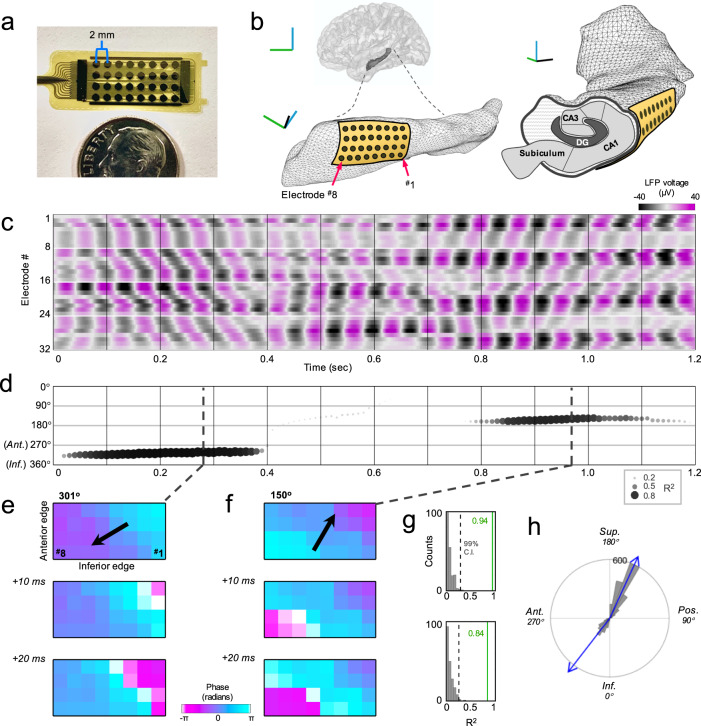
Bidirectional traveling oscillations over the hippocampal surface. **a** Microgrid and connector (left) with dime for scale. **b** Lateral view of left hippocampus within 3D glass brain, and enlarged view (rotated) with approximate size and positioning of microgrid placement (blue line: superior, green: anterior, black: lateral). Coronal cross-section schematic of subfields at right. **c** Filtered LFP around example peak frequency (13.8 ± 1.5 Hz, awake Participant 5) in color scheme (microvolts), white lines separate grid rows. Cycle lags evident across consecutive electrodes suggest spatiotemporal wave travel. **d** Direction of TW in (**c**) calculated every 10 ms (size and shading: regression *R*^2^). In this segment, the oscillation initially travels antero-inferiorly, then directionality declines briefly (low *R*^2^) followed by a robust reemergence in a postero-superior route. **e** Instantaneous phase shown in grid orientation for consecutive timepoints (top panel: dotted line in (**d**)), phase gradient illustrating antero-inferior directionality. **f** Same as (**e**), for example, timepoints with postero-superior directionality. **g** Top and bottom panels show regression model *R*^2^ values (green) for upper panels in (**e** and **f**), respectively, shuffled distributions in gray (dotted line: 99% confidence interval). **h** Circular distribution of directions for this frequency for all baseline period timepoints (10° bins, blue arrows: modes; Inf: inferior, Pos: posterior, Sup: superior, Ant: anterior).

The hippocampal surface is directly accessible during temporal lobe surgery^29^. In this work, we used this unique opportunity to implement a novel recording approach with conformal thin-film microgrid arrays (2 mm spacing, 4 × 8 layout; Fig. 1a), enabling uniform two-dimensional sampling of hippocampal activity in vivo at high density^30^.

## Results

Intraoperative recordings were performed in six participants (Supplementary Table 1) undergoing anterior temporal lobe surgery for medically refractory epilepsy (*N* = 5) or antero-lateral temporal tumor removal (*N* = 1). Four participants had right-sided surgery and were under general anesthesia (unconscious) while two left-sided participants were awake for clinical reasons. All surgeries involved an identical initial approach and exposure of the ventricular hippocampal surface, upon which electrode microgrids were placed approximately over the CA1 region (Fig. 1b).

For each participant, a segment of the recording was selected as a baseline period for further analyses (40–190 s for each participant, selected to avoid signal artifacts and to overlap with behavioral task periods in awake participants). We calculated wavelet power spectra across this baseline recording period for each electrode (‘Methods’) and predominant individual (preferred) frequency peaks were identified from the adjusted spectral profiles for subsequent analyses (‘Methods’; Supplementary Fig. 1), similar to prior study approaches^12,13^. We explored a range of 1–15 Hz and found 13 unique preferred frequencies across all six participants (Supplementary Table 2).

**Fig. 2:**
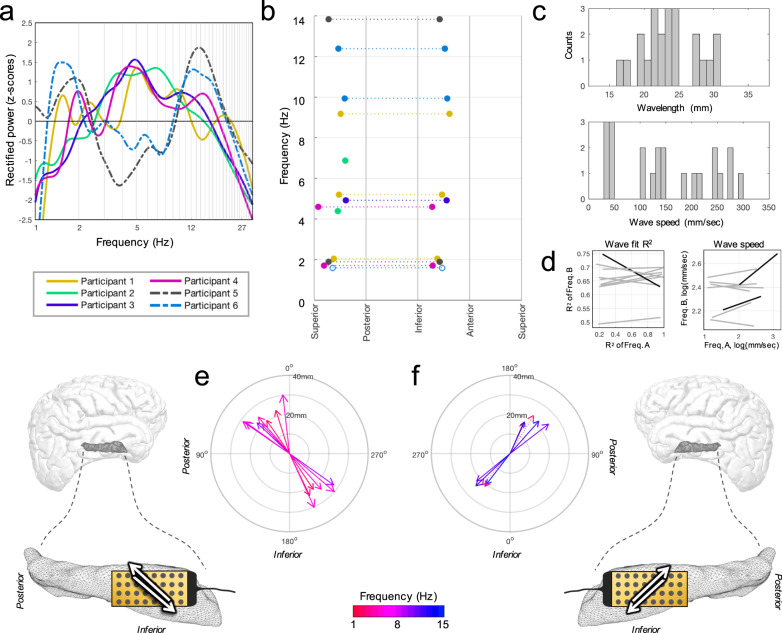
Propagating hippocampal oscillations among all participants. **a** Rectified power spectra as channel means for each participant. Awake participants (left-sided; dotted lines) appeared to have stronger power in delta and alpha ranges while theta appeared stronger in anesthetized patients (right-sided). **b** Direction versus frequency among all participants with consolidated anatomic orientation. Dotted lines connect each bidirectional frequency (open circles: n.s., Hodges-Ajne test). **c** Distributions of wavelengths (top) and speeds (bottom) for all modal directions averaged across baseline periods. **d** Least squares lines (gray) are shown for Spearman correlations of wave fit (*R*^2^, left panel) and speed (right panel) for any two frequencies in the same participant (9 possible combinations; see Supplementary Table 3). Significant combinations (black; >99% CI) were uncommon. **e** TW predominant directions (arrows) for all uni- and bidirectional instances among right-sided participants (color: center frequency, length: wavelength). Supero-posterior and infero-anterior directions predominated, depicted as white arrows in the adjacent schematic (connector orientation in black). **f** Similar for left-sided participants, appearing anatomically mirrored between the hemispheres (compare to (**e**)).

Since consciousness states and laterality influence oscillation frequencies^28,31,32^, we assessed conventional frequency bands in channel-averaged spectra between groups. Mean theta power values across participants appeared higher in the anesthetized (right-sided) participants than the awake (left-sided) participants (group means: −0.321 vs. 0.061 *z*-scores, respectively; Fig. 2a), though the reverse was true for delta (−0.645 vs. 0.967) and alpha bands (0.328 vs. 1.320).

### Traveling oscillations

To investigate whether the predominant frequency peaks identified for each participant propagated across the hippocampal surface, we filtered the local field potential (LFP) around these center frequencies with a 3 Hz bandwidth^13^. Progressive phase offsets across consecutive electrodes were easily observed in many cases (Fig. 1c) suggestive of wave travel across space and time. Two-dimensional (plane wave) regression was applied modeling oscillation phase as a function of distance in the orthogonal septotemporal and transverse axes, methods previously validated on traveling cortical surface waves (‘Methods’)^13,23^. *R*^2^ values were obtained for the goodness-of-fit of the optimal model at each timepoint, along with the estimated wave direction, spatial frequency, wavelength, and speed (‘Methods’; Supplementary Movie 1). Chance distributions for regression models were also created for each timepoint using random permutations of electrode locations (200 iterations).

An example frequency (13.8 Hz) is shown in Fig. 1 (animated in Supplementary Movie 1), in which an antero-inferior direction of propagation is evident for several cycles. However, several cycles later the wave has reversed course, heading in the roughly opposite direction (postero-superior). Both directions featured timepoints with *R*^2^ values well above the 99% confidence interval (CI) of their chance distributions, and a circular distribution of all baseline timepoints exceeding their *R*^2^ 99% CI confirmed that this frequency nearly always propagated along these two routes (though more often postero-superiorly; Fig. 1g, h).

Across participants, all identified oscillation peaks featured TW properties (Fig. 2 and Supplementary Fig. 2). Moreover, most had bimodal circular distributions (Hodges-Ajne test, *p* < 0.001 for timepoints exceeding their shuffled 99% CI; Supplementary Table 2), with 10 of 13 identified as bidirectional TWs. Only one participant appeared to have predominantly unidirectional propagation (Participant 2; for both 4.4 and 6.9 Hz oscillations), directed postero-superiorly.

Across all participants and regardless of hemisphere, oscillations traveled in similar anatomical routes, specifically antero-inferior and/or postero-superior quadrants (Fig. 2b, e, f and Supplementary Fig. 2). Mean wave parameters included spatial frequencies ranging from 11.9°/mm to 21.6°/mm, and wavelengths from 16.7 to 30.1 mm (Supplementary Table 2 and Fig. 2c, e, f). Peak frequencies (ranging 1.6–13.8 Hz across participants) were positively related to their speeds (means ranging 31.4–296.3 mm/s; Spearman’s rho = 0.97, *p* < 0.001 with bidirectional instances pre-averaged; Supplementary Table 2). For any two frequencies in the same participant (9 possible combinations, Supplementary Table 3), timepoint correlations for *R*^2^ (TW goodness-of-fit) and wave speed parameters were uncommon (Fig. 2d), and pairs of oscillations frequently traveled in both similar and different (often opposite) directions.

We repeated the entire analysis above with a variety of subsampled electrode configurations to rule out recording or mechanical artifact on directionality. Relative to shuffled (chance) distributions, actual propagation distributions and modes adhered to their anatomic alignments with little variation (Supplementary Figs. 3 and 4).

As an additional confirmation of the propagation axis, in one participant we exploited the intraoperative ability to experimentally rotate the grid (Supplementary Fig. 5). For Participant 2 in whom unidirectional (postero-superior) theta oscillations predominated, we filtered the data broadly between 3 and 8 Hz and found that manual clockwise rotation of ~25° produced a roughly analogous counterclockwise rotation of the calculated TW angle (32°; *p* < 0.001, non-parametric multi-sample circular median test). Similarly, we performed a pseudo-rotation analysis by subsampling a 3 × 2 grid configuration shifted either −45° or +45°, again finding that anatomic propagation routes were similar (Supplementary Fig. 4e).

Prior studies sampling from only the more septal portion of the structure^15^, or the entire length with more sparse depth electrode placement^12,16^, describe only unidirectional waves traveling toward the temporal pole. Therefore we assessed whether our bidirectional findings were influenced by where the data were sampled from on the hippocampal surface, repeating the above TW analyses after subsampling electrodes to 4 × 4 square layout and advancing anteriorly in 2 mm steps while repeating the analysis on the same timepoints. The antero-inferior and postero-superior quadrants were preserved as dominant bidirectional routes along the hippocampal body (Supplementary Fig. 6). There appeared to be a slight angle shift with a more inferior–superior axis tendency closer to the septal pole and an anterior–posterior axis tendency closer to the temporal pole. This anatomic aspect appeared to follow the curvature of the hippocampal body and contribute to the oblique orientation of the propagation axis, though we were underpowered to assess this further.

### Behavior

If traveling hippocampal waves indeed organize spatiotemporal neural activity flow during cognitive computations, their consistency and predominant routes may be modulated by a behavioral task. Participants 5 and 6 performed a visual naming task in which they were shown a picture of an object and asked to say the name of it as soon as they could, with a variable delay between trials. This task is easily implemented in the operating room and the left hippocampus is known to be activated^33,34^, and potentially relied upon for^35^, visual naming. It provided two time-locked events: stimulus onset (image on a screen) and speech onset (speaking the answer). Median reaction times between these events were 1.68 s (range: 0.67–15.85) and 1.17 s (range: 0.89–1.76) for Participants 5 and 6, respectively.

**Fig. 3:**
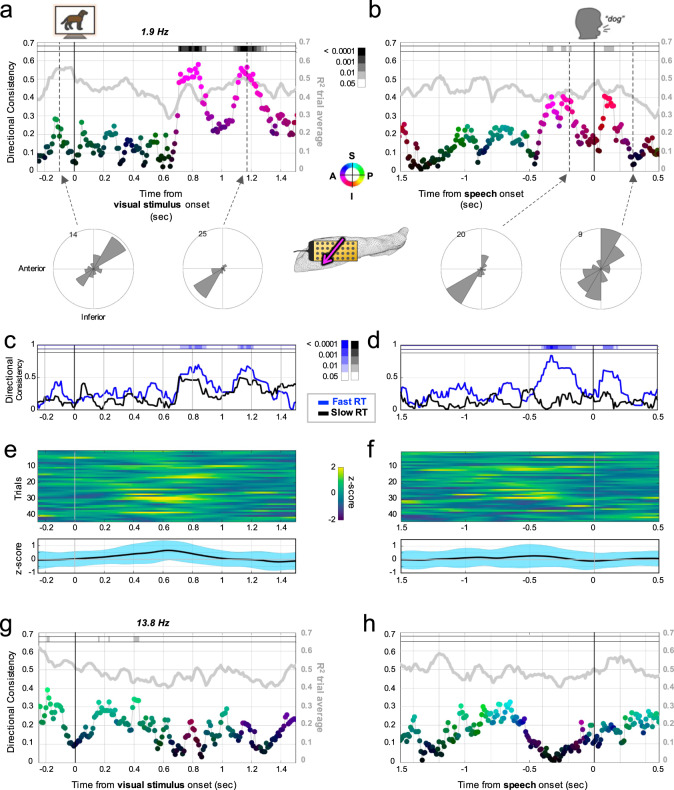
Alignment of propagation direction across trials during a visual naming task. **a** Directionality consistency values (DC; dots) across trials for the 1.9 Hz peak frequency in Participant 5 (*n* = 43 trials) with timepoints locked to the visual stimulus at time zero, colored as average direction of travel (circular mean, saturation proportional to maximum DC, direction according to color wheel: I: inferior, P: posterior, S: superior, A: anterior) over the hippocampal surface (pixels in lane at top: FDR-corrected Rayleigh test *p*-value, *p*_FDR_; see scale bar). The propagation strength across trials was estimated by mean *R*^2^ (gray superimposed line: separate *y*-axis at right) which was roughly similar when trials were aligned in direction or not (high or low DC). Directional distributions are shown below for two example timepoints (dotted lines). Roughly 700 ms after the stimulus waves abruptly begin to align antero-inferiorly across trials (strongest *p*_FDR_ = 3.2^−5^, Rayleigh). **b** DC now locked to speech onset (time zero). Alignment of propagation across trials similar to (**a**) is evident again shortly before speech (strongest *p*_FDR_ = 0.0243, Rayleigh) and resolves back to baseline levels after speech onset. **c** Trials in (**a** and **b**) were grouped by reaction time (fast or slow, median split). Fast reaction time trials increased in DC significantly after the stimulus (strongest *p*_FDR_ = 0.0049, Rayleigh; see scale bars) and (**d**) around speech onset (strongest *p*_FDR_ = 5.6^−6^, Rayleigh), whereas slow reaction time trials did not (empty *p*_FDR_ lanes). **e** Trial rasters of analytic amplitude for the 1.9 Hz frequency described in (**a**), averaged across trials in bottom panel (mean: black trace; blue envelope: standard deviation) with a subtle increase peaking at ~600 ms, returning to baseline as DC in (**a**) begins to increase. **f** Similar to (**e**) now locked to speech onset (time zero) with amplitude staying roughly at baseline during DC changes in (**b**) (mean: black trace; blue envelope: standard deviation). **g**, **h** Illustrate the 13.8 Hz peak frequency (similar to (**a**) and (**b**)) in the same participant with limited DC value trends (strongest *p*_FDR_ = 0.0119, Rayleigh) that dissipate by ~500 ms into trials.

We time-locked trial data to the events above and at each timepoint calculated the instantaneous wave direction for a given peak frequency. We then obtained the circular average across trials (mean resultant length Rayleigh test statistic corrected for false-discovery rate, *p*_FDR_), termed the directionality consistency (DC)^13,36^. For a given timepoint across trials, DC could range from 0, indicating a uniform or chance distribution of directions, to 1, indicating waves traveled in the same direction in each trial. DC could not be assessed in Participant 6 due to few valid trials (*n* < 10) after artifact-related trial exclusions. For Participant 5 (*n* = 43 trials), DC abruptly increased about 700 ms after stimulus onset for her 1.9 Hz peak frequency with an antero-inferior direction (*p*_FDR_ < 0.0001, Rayleigh) and this was also evident around speech onset (*p*_FDR_ < 0.05, Rayleigh; Fig. 3a, b and Supplementary Fig. 7). There was a subtle amplitude increase after the visual stimulus resembling an event-related potential as anticipated^37^ (Fig. 3e, f), though the timing of peak analytic amplitude in a given trial was not related to the timing of peak traveling wave *R*^2^ (Spearman rho = −0.270, *p* = 0.08, *n* = 43 trials). In fact, DC increased only as the mean analytic amplitude receded back toward baseline levels with an inverse timepoint-by-timepoint correlation in the first 2 s after the stimulus (Spearman rho = −0.26, *p* < 0.001).

To assess relevance for performance, we divided the trials using a median split of reaction time. Trials with faster reaction times (better performance) exhibited high DC values prior to speech onset (*p*_FDR_ < 0.0001, Rayleigh), while trials with slower reaction times (including trials where the participant eventually stated they did not know the answer) did not (Fig. 3d). DC trends for the 13.8 Hz frequency were negligible for either stimulus or speech (*p*_FDR_ > 0.05; Rayleigh_;_ Fig. 3g, h).

**Fig. 4:**
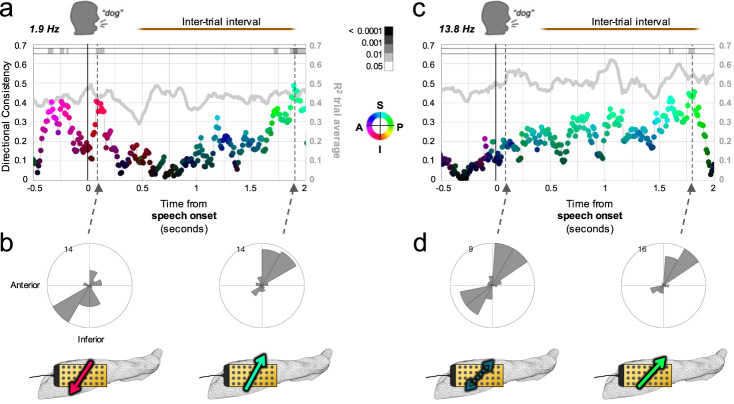
Alignment of propagation direction during inter-trial intervals. **a** Directionality consistency (DC; dots) values for the 1.9 Hz peak frequency in Participant 5 (*n* = 43 trials) locked to speech onset similar to Fig. 3b (same scale bars and direction color wheel), but extending 2 s into the inter-trial interval (gray line: mean *R*^2^ across trials with separate *y*-axis at right; shaded region: DC 95% CI for shuffled timepoints). After speech onset, the alignment across trials toward the temporal pole recedes and then reemerges, but aligned more toward the septal pole as the subsequent trial approaches. **b** Directional distributions for two example timepoints (dotted lines in (**a**), *p*_FDR_ = 0.0243 and 0.0087, respectively, Rayleigh), illustrating the direction reversal. Bottom, anatomic schematics (left hippocampal microgrid, arrow color same as (**a**)) conveying average direction of wave travel over hippocampal surface at those timepoints. **c** Same as a for the 13.8 Hz frequency, with a low DC around speech onset (though usual quadrants predominate) followed by a steady increase in alignment more toward the septal pole as the subsequent trial approaches. **d** Directional distributions and schematics for two example timepoints marked by the dotted arrows in (**c**) (*p*_FDR_ = 0.4583 and 0.0189, respectively, Rayleigh).

Different cognitive operations engage distinct neural circuits, and it has been suggested this could modify phase gradients that produce an observable change in TW directionality^13^. Thus in addition to active visual naming periods, we examined the non-active (or anticipatory) inter-trial intervals between speech and the subsequent trial stimulus onset (median duration: 2.35 s, range: 1.10–8.38). As time progressed during inter-trial intervals, both the 1.9 and 13.8 Hz waves exhibited increasing DC, toward the postero-superior direction (*p*_FDR_ < 0.05, Rayleigh; Fig. 4 and Supplementary Fig. 7). Thus overall, both frequencies were modulated but differentially: the 1.9 Hz oscillation waves consistently reversed directions between the active trial and non-active (or anticipatory) inter-trial portions of the task, and the 13.8 Hz wave showed alignment across trials only during the inter-trial portion.

### Propagation direction prediction

**Fig. 5:**
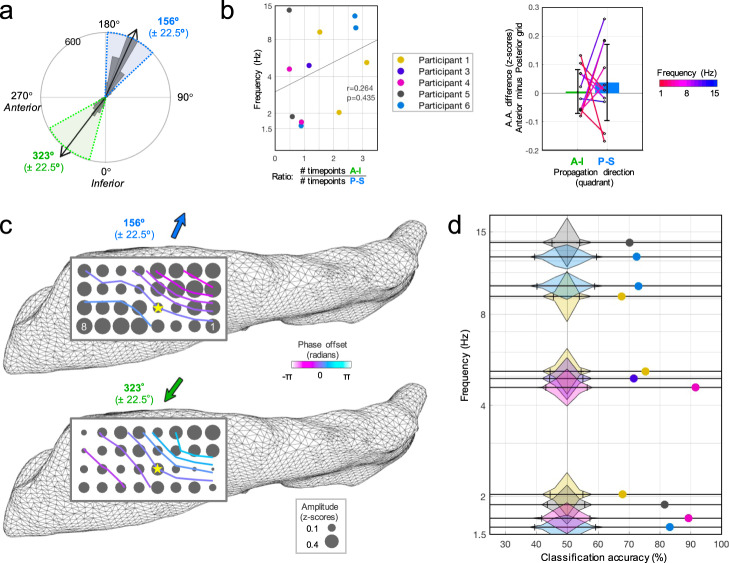
Topographic pattern of oscillation strength predicts propagation direction. **a** Example of angle split procedure for the distribution of bimodal TW timepoints (Participant 5, 13.8 Hz oscillation). Timepoints having wave directions within a 45° spread of either mode were grouped into separate conditions. **b** Across participants, the frequency (*n* = 11) of a predominant (peak) oscillation did not predict whether it tended to propagate toward the temporal or septal pole more often, assessed by the ratio of total timepoints in each direction condition (left panel; *p* = 0.44, Spearman correlation, A-I: antero-inferior direction, P-S: postero-superior direction). Analytic amplitude (A.A.) differences between the anterior and posterior halves of the grid for these same frequencies (colored lines; compiled across participants) were not different between the two directions (right panel; *p* = 0.57, two-sided paired *t*-test; mean ± standard deviation). **c** Average channel amplitudes (dot size: mean *z*-score) across all timepoints split by condition, for example, frequency in (**a**), suggestive of distinct surface activity topography for each propagation direction. Contour lines overlaid are interpolated estimates of phase gradient offsets (circular mean across all condition timepoints) along the surface relative to electrode #12 (yellow star). **d** Probability density envelopes (10,000 iteration shuffled distributions, 4% bins) for SVM model classification accuracy among all peak frequencies across participants (chance: 50%, hatch marks: 99% CI) and actual/observed values (colored dots; see legend in (**b**)).

Do directional changes reflect distinct circuits that drive specific propagation routes when they engage? Such spatial structure could be evident as distinct areas on the hippocampal surface oscillating more (or less) strongly, or at certain frequency ranges, for different TW directions as suggested by coupled oscillator theories^13,28^. For each frequency with a bimodal directional distribution, we selected all timepoints having a calculated TW direction within 45° of each mode, forming two groups (Fig. 5a). The proportion of timepoints going toward the temporal pole (anterior-inferior) versus the septal pole (posterior-superior) was not related to frequency (Spearman rho=0.264, *p* = 0.44; Fig. 5b), and oscillation amplitude differences between the anterior (16-electrode mean) and posterior (16-electrode mean) halves of the grid were not different between the two directions (*p* = 0.57, paired *t*-test; Fig. 5b). However, distinct topographic patterns appeared evident across the grid when averaging channel amplitudes for each directional group (Fig. 5c), suggestive that such mesoscopic detail may hold predictive value. The electrodes’ corresponding oscillation amplitudes for these timepoints were used as features to train a support vector machine (SVM) classifier model to predict which direction the wave was traveling at a given timepoint. We used randomly drawn timepoints comprising equal group sizes to train the model with 5-fold cross-validation (80% train, 20% test). Prediction accuracy surpassed the 99% CI (shuffled group labels, 10,000 iterations) in all 11 bimodal instances (Fig. 5d and Supplementary Table 4).

## Discussion

Using novel microgrid recordings over the human hippocampal surface, we demonstrate that low-frequency oscillations dynamically reverse course along postero-superior and antero-inferior routes with respect to the structure’s longitudinal axis. These properties were mirrored across participants, hemispheres, consciousness states, and preserved anatomically when changing electrode configurations (Fig. 2 and Supplementary Figs. 2–4, 6) and when manually rotating the grid (tested in one participant; Supplementary Fig. 5), suggesting they represent typical propagation properties of the human hippocampus.

Since low frequencies are largely phase-invariant as one moves superficially from the stratum pyramidale^15,16^, electrodes presumably recorded local CA1 activity given the positioning of the grid on the ventricular hippocampal surface (Fig. 1b). TW propagated obliquely along both septotemporal and transverse (CA3–CA1–subiculum) axes, perhaps in part due to the curvature of the hippocampus under the full rectangular grid in our models (Supplementary Fig. 6). However, this could also suggest that propagation toward the temporal pole tends to engage CA3-to-CA1-to-subiculum activity flow (the classic feedforward circuit), whereas propagation toward the septal pole might engage a known reversal of this circuit^18^. Further study coupled with wider sampling (CA3, subiculum), and perhaps simultaneous depth electrodes for 3D laminar sampling could investigate such concerted flow patterns.

Bidirectional TWs have been described in the cortex of primates and humans^19,22^, and similar directional versatility of low-frequency oscillations in the hippocampus may reflect information flow as distinct circuits engage and disengage. In other words, networks connected at different levels of the hippocampal body may generate oscillations that produce unique phase gradient changes (directional shifts) when activated. The spatial migration of intrinsic hippocampal oscillators, or even reversals in circuit flow, could also drive phase gradient changes^18,38^. The presence and directionality of hippocampal TWs were indeed modulated during distinct cognitive stages of a behavioral task, evident as dynamic alignment of ongoing oscillations across trials relative to task events, though this was assessed in only one participant (Participant 5 in Figs. 3 and 4; Participant 6 lacked sufficient task data due to limitations from signal artifact). Furthermore, machine learning models trained on the topographic patterns of oscillation strength over the surface could predict which way an oscillation was traveling (Fig. 5). Directions of propagation may therefore act as biomarkers for distinct hippocampal network operations. The proportions of time in each direction of propagation varied across participants and frequencies (Fig. 5b) and appeared modulated during behavior, and so such proportions may reflect related factors such as the balance of cognitive or attentional states as suggested by the behavioral results, the depth of anesthesia, or perhaps even the degree of disease.

The septal and temporal hippocampal portions interface with multiple brain systems subserving different information content^1–3^, yet rely on the hippocampus to parse and integrate this information to support memory and other cognition^4–6,39^. Recent work has noted a greater prevalence of faster (theta) frequencies in the septal pole and slower (delta) frequencies in the temporal pole, plausibly supporting spatial and non-spatial processes, respectively^28^. Coupled oscillator models would suggest theta oscillations would tend to flow from faster to slower frequency sites or from sites with stronger oscillations. While we did not observe these relations (Fig. 5b), the correlation between wave frequency and speed here is nevertheless suggestive of coupled oscillator properties. Moreover, the prediction of direction by distinct topographic amplitude patterns along the surface (Fig. 5c, d) suggests that mesoscopic network interconnections along or across the structure (i.e., not solely relative differences at the poles) drive bidirectional oscillations that migrate from one region and influence (and potentially configure) the activity in another region^11–16,40^.

We therefore speculate that bidirectional TWs may enable versatility for routing and weighting different information types during memory or other cognitive processing. In other words, in the hippocampus this ordered framework could suggest that while waves moving anteriorly are pre-weighted with detailed visuospatial information that may then integrate with some amount of emotional information^7,8^, waves moving posteriorly could be weighted more strongly by emotional information that is then tagged with visuospatial content. Further behavioral investigations of dynamic directionality comparing different task modalities are necessary to investigate this potential functional relevance.

Clinical depth electrodes provide crucial windows into the neurophysiology of deep structures, but offer only a handful of intra-hippocampal recording sites in humans (Supplementary Fig. 8). Single probes are unable to measure in orthogonal dimensions, further limiting the estimates of directionality and other properties such as wavelength and speed. Finally, oscillation phases, upon which TW calculations often rely, change up to a half-cycle as penetrating electrodes simply traverse certain hippocampal lamina including the strati pyramidale and radiatum^15,16,41^. To combat these factors, we leveraged a microgrid conformed to the largest hippocampal portion in humans, the anterior-intermediate aspect^24^. We accounted for a full 360° of phase gradients, instrumental for the discovery of diverse directionalities of TWs in the cortex, with 2.5× the sampling density of standard human depth recordings to improve spatial wave resolution^12,13,20^. Higher density electrodes could reveal more complex paths at even finer scales^11,19^.

We suspect bidirectional propagation may be common to the majority of the hippocampal axis, since similar routes persisted along the hippocampal body (Supplementary Fig. 6). However, prior studies sampling the septal pole more heavily than our coverage^12,15,16^ instead concluded that theta waves travel unidirectionally (posterior to anterior). Given the differences in topographic connectivity, it is plausible the septal pole conducts a prominent unidirectional generator while the temporal pole conducts bidirectional generators. High-density recordings uniformly sampling the entire length of the human hippocampus could resolve this important mesoscopic question.

We targeted the dominant frequency peaks unique to each participant, since human hippocampal frequencies differ from animals, vary between and within individuals, and depend on a subject’s state of awareness (Fig. 2a), among other factors^12,27,31^. All prominent low-frequency oscillations in our study exhibited TW properties, not just those at or near the canonical theta band. Multiple peak frequencies in the same participant (Fig. 2 and Supplementary Fig. 2) may reflect interactions with different cerebral networks conducting independently (Fig. 3) along a common hardwired hippocampal thoroughfare^13,22,28^ embedded with machinery for integrative (multimodal) neural processing.

Broadening from a unidirectional to bidirectional propagation interpretation has strong implications for mechanistic theories of hippocampal-dependent cognition. This includes the anatomic timing of phase-encoded neural firing (and perhaps relevance for forward or reverse replay sequences), and the question of whether hierarchical cognitive processing sequentially follows this topographic spread^7,8,42,43^. Future TW studies coupled with single-unit recordings and behavior will be crucial to assess how such spatiotemporal computations may support the remarkably flexible cognition integration performed by this structure for diverse brain systems.

## Methods

### Data collection

The experimental protocol was approved by the UCSF Institutional Review Board. Six participants were included in the study (Supplementary Table 1) and all provided written informed consent. Five had medically refractory seizures requiring surgical management due to temporal lobe epilepsy, for which the anterior lobe was resected from a lateral approach, eventually accessing the lateral ventricle and exposing the ventricular hippocampal surface. The other participant (Participant 5) had a circumscribed antero-lateral temporal lobe tumor that required a similar anterior temporal surgery, and resultant exposure of the hippocampus which was presumably normal (unaffected on MRI and intraoperative visual inspection). Intraoperative monitoring was performed as part of routine clinical care, with the experimental use of grids with high-density inter-electrode spacing (see below) instead of standard clinical grid or strip spacing. The recordings occurred under general anesthesia (combinations of dexmedetomidine, propofol, and fentanyl) in Participants 1–4. Participants 5 and 6 were in awake state during the recordings due to clinical factors (intraoperative stimulation mapping procedures) and participated in a behavioral task.

We utilized a 32-contact microgrid manufactured through micro-electro-mechanical system technology by Lawrence Livermore National Laboratories (Fig. 1a), configured as a polymer-metal-polymer stack. Electrodes were cosputtered thin film 60/40 platinum/iridium on gold traces (1.22 mm diameter, 2 mm center-to-center) in a 4 × 8 two-dimensional layout (approximately 110 mm^2^ coverage). Additional rectangular contacts uniformly lined the perimeter of the recording contacts (Fig. 1a) and were shorted together for use as a single recording reference with no subsequent re-referencing (to ensure the original amplitude and phase data were maintained^44^). Briefly during intraoperative downtime, the microgrid was carefully placed parallel to the long axis on the lateral hippocampus (its ventricular surface; Fig. 1b) to achieve conformal surface contact. Irrigated gauze was placed over top to ensure stability during the subsequent recordings. The cable connector edge was always positioned anteriorly toward the temporal pole regardless of laterality.

Referential recordings were obtained using a multichannel amplifier (Tucker Davis Technologies using TDT OpenEx software 2.31) with a fiber-optic connection digitized at 24.4 kHz, for approximately 8–22 min per participant and divided into 2–8 recording blocks for data handling purposes or to separate adjustments in grid placement. Contacts that extended just beyond the hippocampal surface, or were otherwise noted to have low signal or excessive artifact, were excluded from analysis (0–4 channels per participant).

### Signal processing and analysis

All analyses were performed in Matlab (Natick, MA) version R2019a. For each participant, the segment of the recording selected as a baseline period was screened to be largely artifact-free. Segments with epileptiform discharges or artifact (excessive cable movement, electrode pops, etc.) were identified using automated line-length detection methods^45^ followed by manual screening by a trained epileptologist (J.K.), and these segments were excluded from all analyses.

To identify unique oscillation peaks in spectral profiles, each recording channel was pre-filtered (255 Hz low-pass) then downsampled to 512 Hz. Each participant’s recording underwent Morlet (Gabor) wavelet transformation of the entire recording block with 211 logarithmically spaced frequencies between 1 and 32 Hz using the cwt.m function file in Matlab (voices per octave: 42). Transformed data (power, or analytic amplitude and phase using Hilbert transform below) was further down sampled to 30 Hz (greater than twice the highest TW frequency studied) except for illustration and behavior data (Figs. 1, 3, and 4; 100 Hz, and Supplementary Movie 1; 512 Hz).

Power spectra averaged across timepoints illustrated the expected aperiodic power-law trend, along with prominent periodic peaks above this spectral background (Supplementary Fig. 1). A robust regression was fit to the 1–32 Hz log–log spectral trend, subtracted, and the result was *z*-scored. This adjusted power spectrum was assessed for oscillation peaks emerging >1 standard deviation (Supplementary Fig. 1b), and if at least five electrodes on the grid shared peaks within a 2 Hz bandwidth, their averaged peak frequency was deemed a preferred center frequency for that participant (Supplementary Fig. 1c, d), following prior work^12,13^.

### Traveling oscillation analysis

Continuous phase data were obtained for each identified peak frequency above using the Hilbert transform with a surrounding 3 Hz (±1.5 Hz) bandwidth window to account for dynamic peak center fluctuations^13^. For each timepoint, the grid location (*x* and *y* coordinates; independent variables) and phase (dependent variable) for each electrode were fitted to a circular-linear (two-dimensional) regression model^13,20,23^. The model was iterated for spatial directions in 1° steps over the 360° range, and for spatial frequencies in steps of 1°/mm up to 90°/mm based on the spatial Nyquist frequency of our 2 mm inter-electrode grid spacing. During periods with weak or fragmented oscillations across channels, phase analysis can be at risk for spurious findings^46,47^. To counter-act this, we first averaged the analytic amplitude across all channels and excluded timepoints with the lowest 10% of values, then imposed a minimum of 3 continuous cycles of the oscillation to permit timepoint inclusion.

Hippocampal and cortical TWs are known to be roughly linear in offset over space and are therefore approximated well by a planar phase gradient^15^. A circular-linear model of oscillation phase as a function of distance in the orthogonal septotemporal and transverse axes was generated for each timepoint to obtain the directionality and *R*^2^ for its goodness-of-fit (offset of phase residuals from the model prediction; Supplementary Movie 1), methodology adapted from Zhang et al.^13^. We also obtained the spatial frequency (repetency, or change in fitted phase in radians per millimeter), wavelength (2π divided by spatial frequency), and wave speed (product of wavelength and frequency)^13^. Shuffled distributions were also obtained for each timepoint by randomizing the electrode locations with 200 iterations, and a timepoint was deemed to have a valid TW if the observed *R*^2^ value was greater than the one-sided 95% CI (Fig. 1g).

The calculated TW directions from all timepoints meeting the threshold criteria above were binned in 10° steps plotted as circular distributions to illustrate any preferred directionality (Supplementary Fig. 2); a null assumption would be a uniform distribution across all angles (0–360°)^36^ or by extension within the confidence bounds of distributions from shuffled electrode positions^48^. Distributions were formally considered bidirectional (bimodal) for statistical testing purposes if the smaller peak angle was at least 25% of the height of the largest peak after smoothing (see below), in which case a Hodges-Ajne test was used to test circular non-uniformity (*p* < 0.001 considered requisite), whereas a Rayleigh test was used for unidirectional instances^36,49^. Both are tests for angular uniformity among data in circular distributions as opposed to one- or two-sided tests on linear data. The predominant directions for all uni- and bi-directional cases were inferred as the modes obtained after applying a 60° circular smoothing window^50^.

To assess different electrode layouts, we performed the same analysis as above (TW models, distributions, and mode calculations) after including only select electrodes for each iteration illustrated in Supplementary Figs. 3, 4, and 6. We utilized the same valid timepoints identified from the full grid analysis (again defined as wave fit *R*^2^ > 99% CI during at least 3 continuous cycles above our 10% amplitude threshold) to enable direct comparison of our main analysis to these subsampled electrode configurations.

For the experiment in which the grid was manually rotated, the positions of Participant 2 and the intraoperative camera were fixed in place allowing estimation of the physical angle of manual rotation from intraoperative photos. Using superimposed lines lengthwise along the grid center for each condition, we calculated relative angles using their slope arctangents (Supplementary Fig. 5). A 3–8 Hz bandwidth was used to encompass both unidirectional theta frequencies in this participant and distributions were created following the above procedures for baseline recording periods in each of the two conditions (standard anterior-posterior and rotated orientations). The angular difference was obtained from their circular means and to assess whether they were significantly different we applied a circular non-parametric multi-sample test to their angular distributions^49^.

### Propagation direction

We assessed whether a bidirectional TW was traveling in one of its prominent directions versus the other based on frequency, amplitude gradients, or the topography of oscillation amplitude over the hippocampal surface. For each bidirectional TW (all instances except the predominantly unidirectional frequencies in Participant 2; see Supplementary Fig. 2), we took all valid timepoints in which the wave was traveling within 45° of either of its two preferred angles (again excluding timepoints with *R*^2^ values less than their own 99% CI), the direction being the binary class label (condition) for each data point. We utilized the entire baseline period to balance out adjacent timepoint similarities; total timepoints for each angle (note: resampled to 30 Hz sampling rate, see above) are provided in Supplementary Table 4. We compared the proportion of timepoints going toward/away from the temporal pole (ratio) by frequency using a Spearman correlation. We compared anterior-posterior amplitude gradients (mean *z*-scored amplitude across timepoints from the 16 electrodes in the anterior half of the grid minus the same from the posterior half) between the two direction conditions using a paired *t*-test (11 instances).

We trained a 5-fold cross-validated SVM model (fitcsvm.m function file in Matlab) using the corresponding amplitude on each electrode as predictors (i.e., up to 32 features). To avoid overfitting, we verified models had far more observations than features (Supplementary Table 4), avoided hyperparameter optimization, and utilized 5-fold cross-validation. Amplitude (similar to the power, or strength) of the frequency at any given timepoint is independent of its phase, and only the latter was used to calculate the TW angle. Therefore, SVM models specifically evaluated whether the pattern of relative activity strength across electrodes (amplitude-based and potentially reflecting local mesoscopic network activity) was predictive of which direction the wave would travel (phase-based). After model training using 80% of data points (randomly chosen), the remaining 20% of data were introduced and tested, and the prediction accuracy was the percentage of correctly predicted labels (TW directions) in this test data. This value was considered significant if it exceeded the 99th percentile of a chance distribution (10,000 iterations of the same procedure during which the original labels were randomly shuffled).

### Visual naming task

Two participants (Participants 5 and 6) were awake during the recordings and performed a visual naming task, which has been associated with left hippocampal activation and potentially reliance^33–35^. In addition to being commonplace in the intraoperative setting for clinical language mapping purposes, the ease of initial instruction and rapid pace of trials also facilitated behavioral data acquisition during our limited-duration recordings. Visual stimuli were pictures of objects^51^ displayed on a laptop monitor via Microsoft PowerPoint (version: 2010) presentation equipped with a simultaneous acoustic tone (exactly 440 Hz) at onset to enable audio data (24 kHz sampling) including speech (microphone near participant’s mouth) to be time-locked with the neural data (same amplifier). The audio recording thus provided a unified solution for marking both stimulus and speech onsets through automated detection of the audio envelope with subsequent manual verification using the audio recording spectrogram. This setup utilized available intraoperative equipment for logistical reasons, though it required post hoc adjustment to adjust for temporal precision. A photodiode on the same laptop screen was used to detect the true visual onset versus the stimulus audio tone, and we shifted the stimulus onset timing accordingly for the behavior analysis in Fig. 3 by the median delay (approximately 272 ms, standard deviation 26 ms). Participant responses in the visual naming task were transcribed from their native language (Vietnamese and Spanish for Participants 5 and 6, respectively) and verified by native speakers. Despite the participants being awake for clinical language mapping purposes, no electrical stimulation occurred during these recordings. For trial-based data, Hilbert-transformed amplitude (*z*-scored within-channel across the entire recording block) and phase were downsampled to 100 Hz and partitioned for each trial by aligning to a window of up to 2 s before and after each event (stimulus or speech onsets), followed by TW regression models at each timepoint, similar to above. Trials in which any electrical or epileptiform artifact occurred were excluded. The DC *p*-value statistic was corrected for false-discovery rate (*p*_FDR_, Rayleigh) using the Benjamani-Hochberg procedure^52^.

### Reporting summary

Further information on research design is available in the Nature Research Reporting Summary linked to this article.

## Supplementary information

Supplementary Information

Description of Additional Supplementary Files

Supplementary Movie 1

Reporting Summary

## Data Availability

The de-identified data that support the findings of this study are available from the corresponding author on reasonable request. Source data are provided with this paper.

## Source data

Source Data

## Code availability

Data processing and analyses were performed in Matlab R2019a, and all code is available from the corresponding author upon reasonable request. Custom automated-detection software for artifact (electrical, or interictal discharge) is publicly available from the Kleen Lab (https://github.com/Kleen-Lab), and circular-linear regression analysis was adapted from publicly available code from the Jacobs Lab (http://github.com/jacobslab/Traveling-wave-analysis). Hippocampal mesh reconstructions for illustrations were obtained from publicly available template brain structures from Freesurfer (https://surfer.nmr.mgh.harvard.edu/).
