# Supplementary material for: Bidirectional propagation of low frequency oscillations over the human hippocampal surface
Source: Nat Commun. 2021 May 12;12:2764. doi: 10.1038/s41467-021-22850-5 (PMC8115072; doi:10.1038/s41467-021-22850-5)
Supplement: Supplementary file 1 — Supplementary Information [file 41467_2021_22850_MOESM1_ESM.pdf]

Supplementary Information

Bidirectional propagation of low frequency oscillations over the human hippocampal surface

Kleen et al.

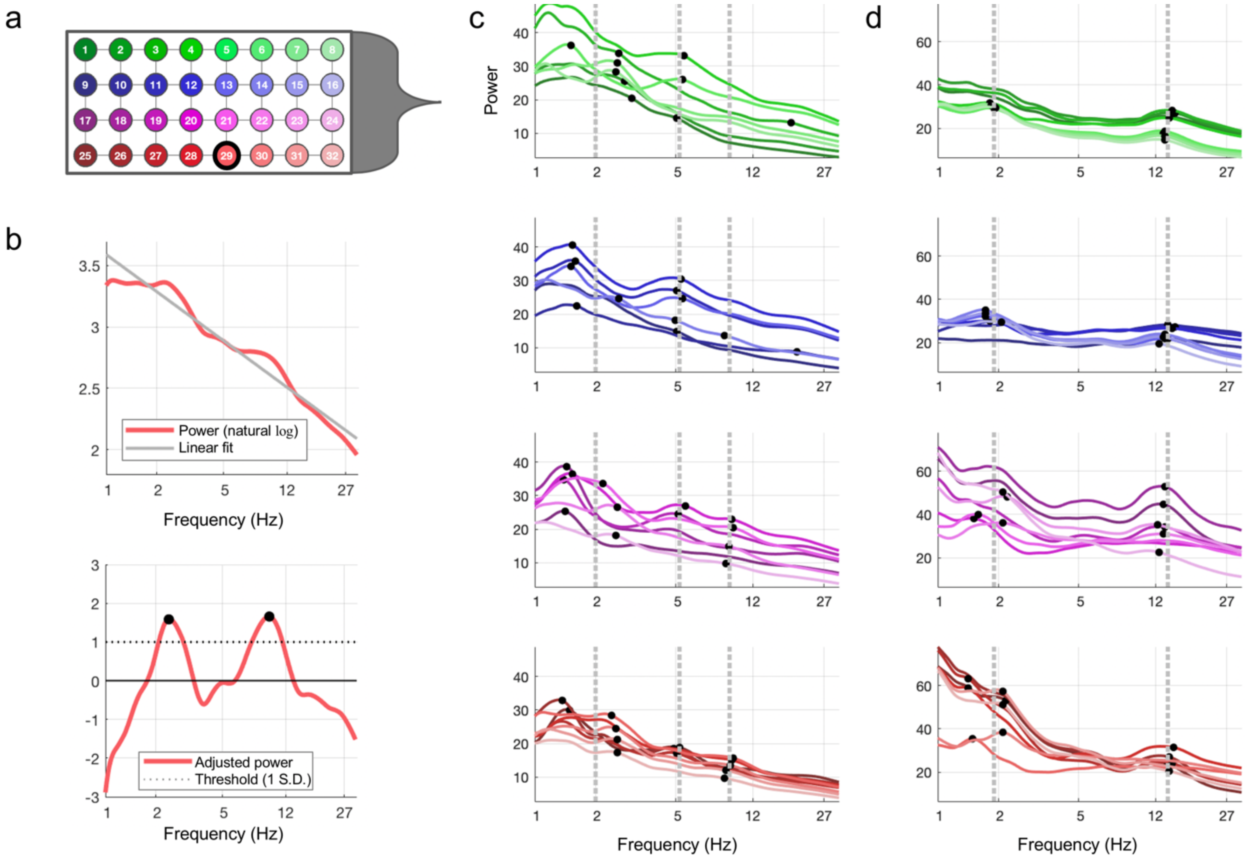

Supplementary Fig. 1.
Spectral composition of hippocampal microgrid recordings. **a** Schematic of microgrid channel layout. Channels 8, 16, 24, and 32 (near connector, grey) were always oriented toward the temporal pole. **b** Wavelet power spectrum of electrode #29 (outlined in a) in Participant 1, averaged across the baseline recording segment and shown in natural log scales. The rectified power spectrum (lower panel) is obtained by subtracting a fitted linear regression, and this adjusted power is z-scored. Black dots mark detected frequency peaks (>1 standard deviation (S.D.) above 1/f rectified spectrum; Methods). **c** Power spectra (linear *y* scale) for all channels from Participant 1 (anesthetized). Each panel shows one grid row, colors according to a, detected peaks as black dots from method in b. Detected frequency peaks are again marked as black dots, and grey dotted lines mark estimated peak frequencies consistent across multiple electrodes (Methods). **d** Same as b for Participant 5 (awake). Note the differences in spectral peaks, largely delta and alpha ranges, compared to more theta-range peaks in c.

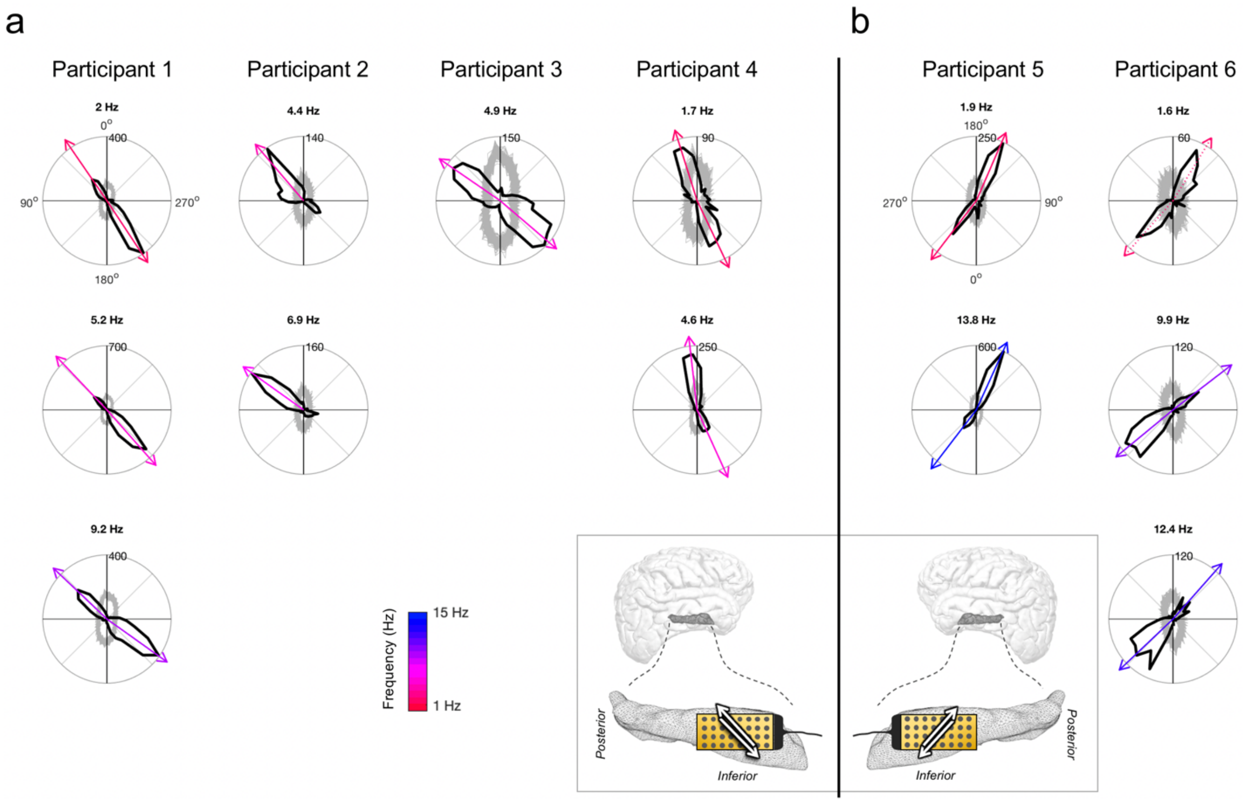

Supplementary Fig. 2.
Individual distributions for each peak frequency. **a** Among right-sided participants, circular distributions (black traces, 10° bins) of TW angles are displayed for all timepoints that surpassed their R^2 99% CI (Methods). Individual participants are in columns and their identified peak oscillations are in rows. Chance distributions (200 for each frequency) from shuffled timepoints are plotted as grey traces. Predominant directions (smoothed circular modes) depicted as arrows for each distribution (summarized in Fig. 2e,f). Lower right schematic shows anatomic orientation and dominant general routes. **b** Same as a for left-sided participants, including chance distributions (200 for each frequency). Dotted lines indicate lack of adjusted directional statistical significance (see Supplementary Table 2).

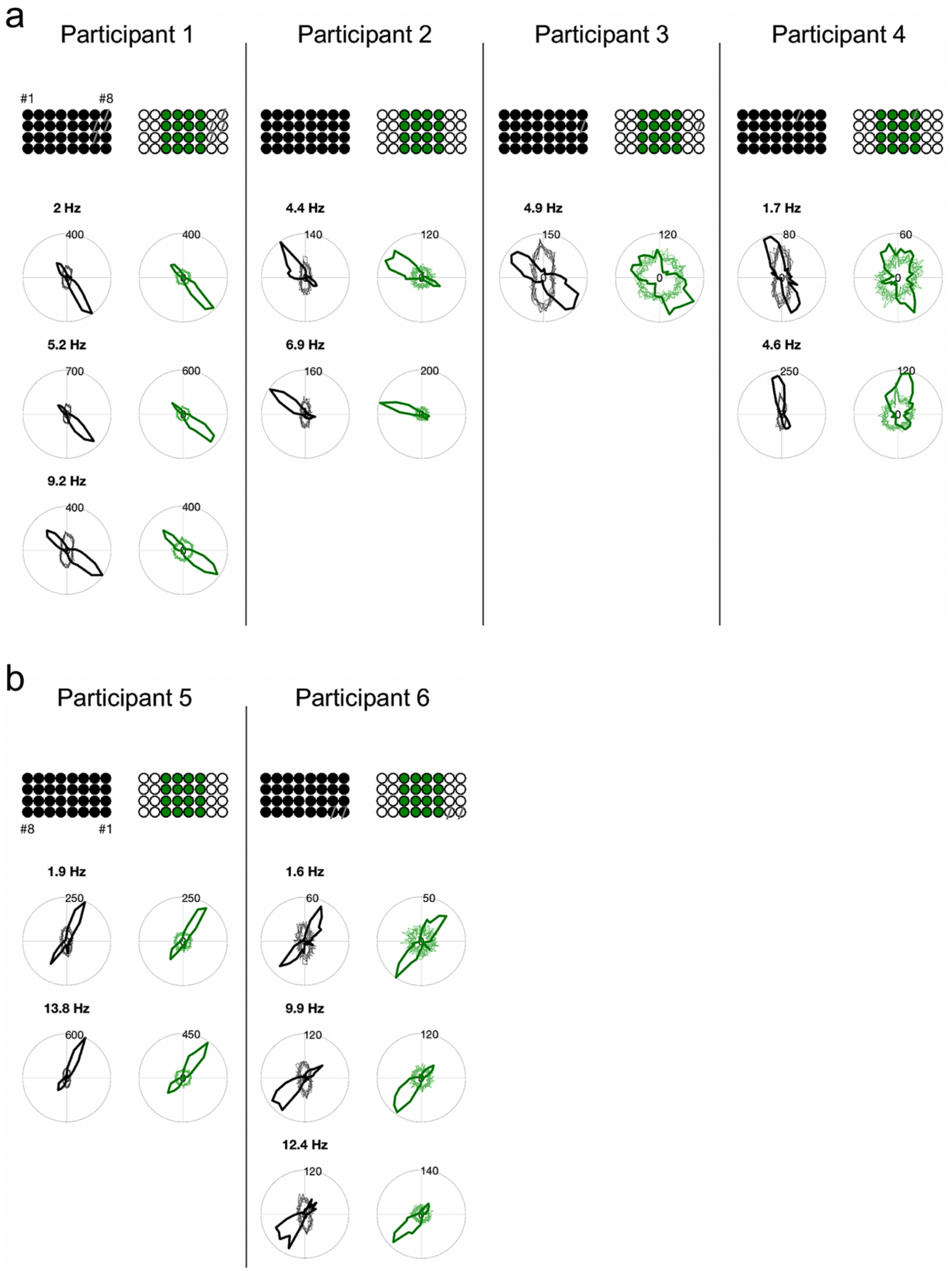

Supplementary Fig. 3.
Electrode layouts using rectangular vs. subsampled square grids show similar predominant propagation directions. **a** Electrode configurations (filled circles in top panels) and resulting propagation distributions for each frequency (lower panels) among all right-sided participants (under anesthesia). Propagation distribution results are shown from the original 4x8 grid (left

panels, black distribution traces, identical to Supplementary Fig. 2A including anatomic orientation; grey traces are 10 example chance distributions) vs. a 4x4 subsampled square grid (right panels, green traces; light green traces are 10 example chance distributions). Both distributions were calculated using timepoints meeting threshold criteria for the original 4x8 grid, thus not all timepoints in the 4x4 square grid analysis may have met our threshold criteria, potentially adding variability. Electrodes with grey hatches were omitted due to connection artifact. **b** Same as a for left-sided participants (awake). Anatomic orientation as in Supplementary Fig. 2b.

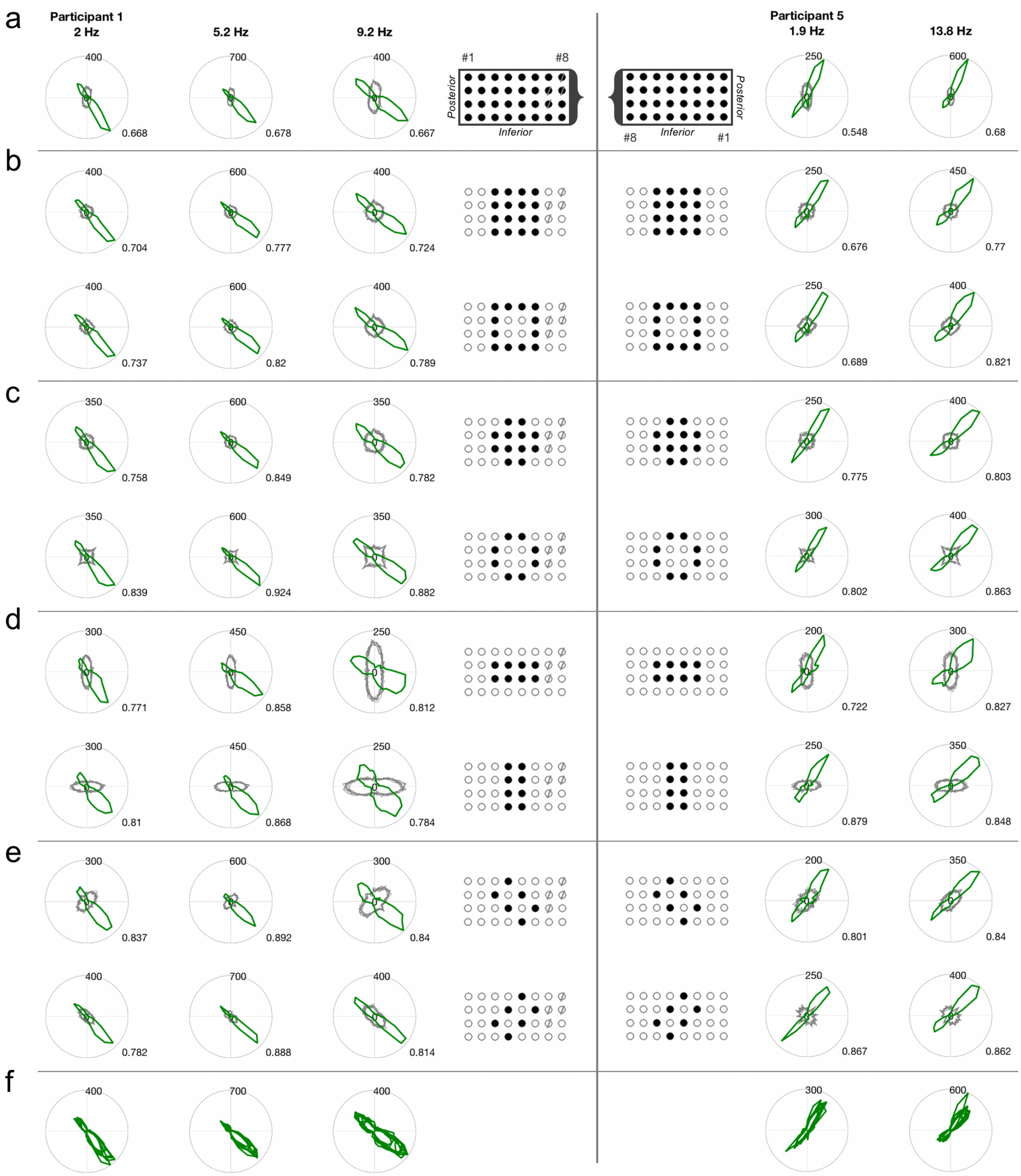

Supplementary Fig. 4.
Preservation of predominant propagation axis across various electrode configurations. **a** Propagation distributions (green traces; grey traces are 10 example chance distributions; average R^2 across timepoints at lower right) from two example participants (left panels, under anesthesia and R-sided; right panels, awake and L-sided) using all available electrodes (identical to Supplementary Fig. 2 and Supplementary Fig. 3 left panels). Electrodes with grey

hatches were omitted due to connection artifact. **b** Same analysis repeated on the timepoints from a using a subset of electrodes comprising a full square configuration (identical to Supplementary Fig. 3 right panels) and separately its perimeter (open circles: omitted). **c** Same analysis using an octagon configuration and separately its perimeter. **d** Same analysis using a small rectangle configuration oriented anterior-posterior and separately when oriented inferior-superior. **e** Same analysis using a small rectangle configuration pseudo-rotated clockwise 45°, and separately pseudo-rotated rotated counterclockwise 45°. **f** Distributions from a-e superimposed. Despite mild variability (e.g. with smaller coverage area, and fewer electrodes which also influenced R^2 as the calculation denominator), the predominant bimodal routes of propagation across the hippocampal surface are preserved regardless of electrode configuration. Note that electrode #1 is in posterior-superior corner in Participant 1 and in posterior-inferior corner in Participant 5, supporting an anatomical orientation of these propagation directions as opposed to a mechanical (equipment artifact) orientation.

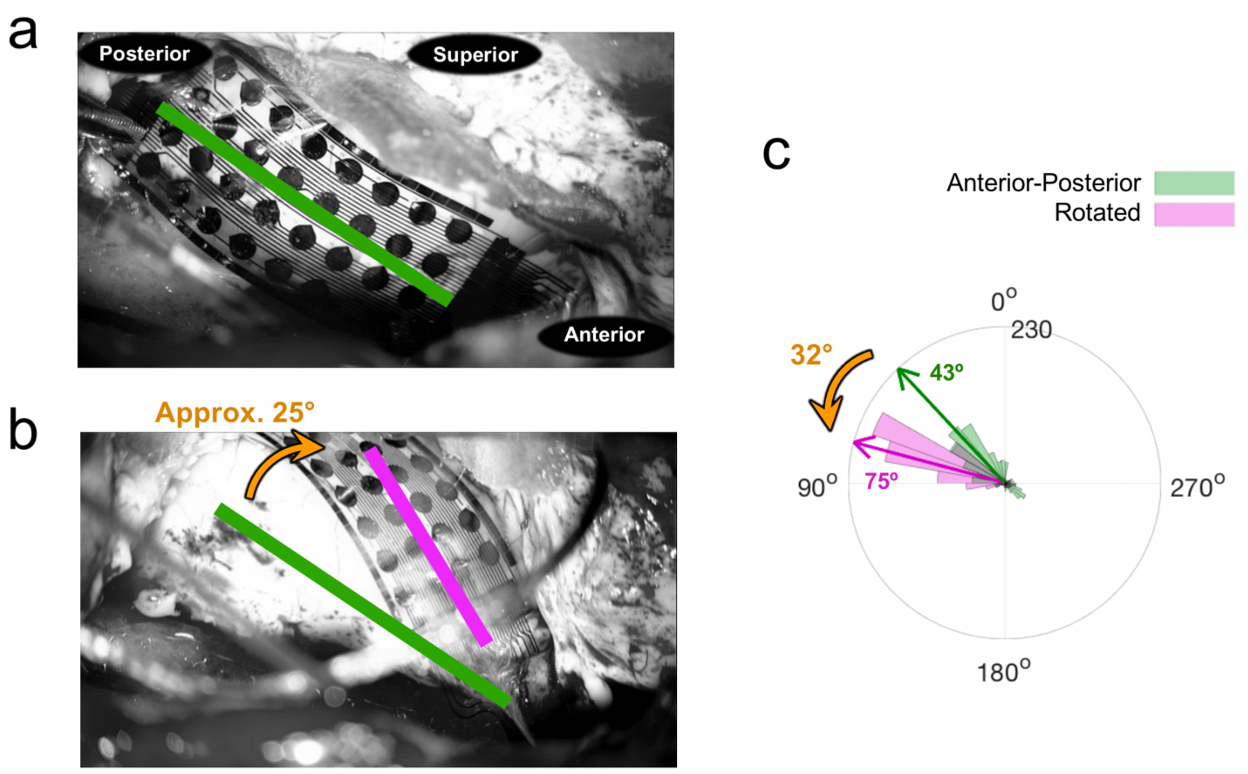

Supplementary Fig. 5.
Consistency of anatomic directionality after manual rotation. **a** Photo of standard anterior-posterior positioning of the microgrid in Participant 2 oriented along the long axis of the right-sided hippocampal surface. Orientation angle approximated by the green line (connector positioned anteriorly). **b** Rotated (clockwise) positioning for a separate recording in the same participant and magenta line approximating this new angle, with arctangents calculated as a manual rotation of approximately 25°. **c** Circular distributions of TW (3-8 Hz bandpass) directions for data recorded during conditions a and b. Mean angles were 43 and 75° respectively, corresponding to a comparable computed counter-rotation of 32° counter-clockwise ($p<0.001$, non-parametric multi-sample test for equal medians).

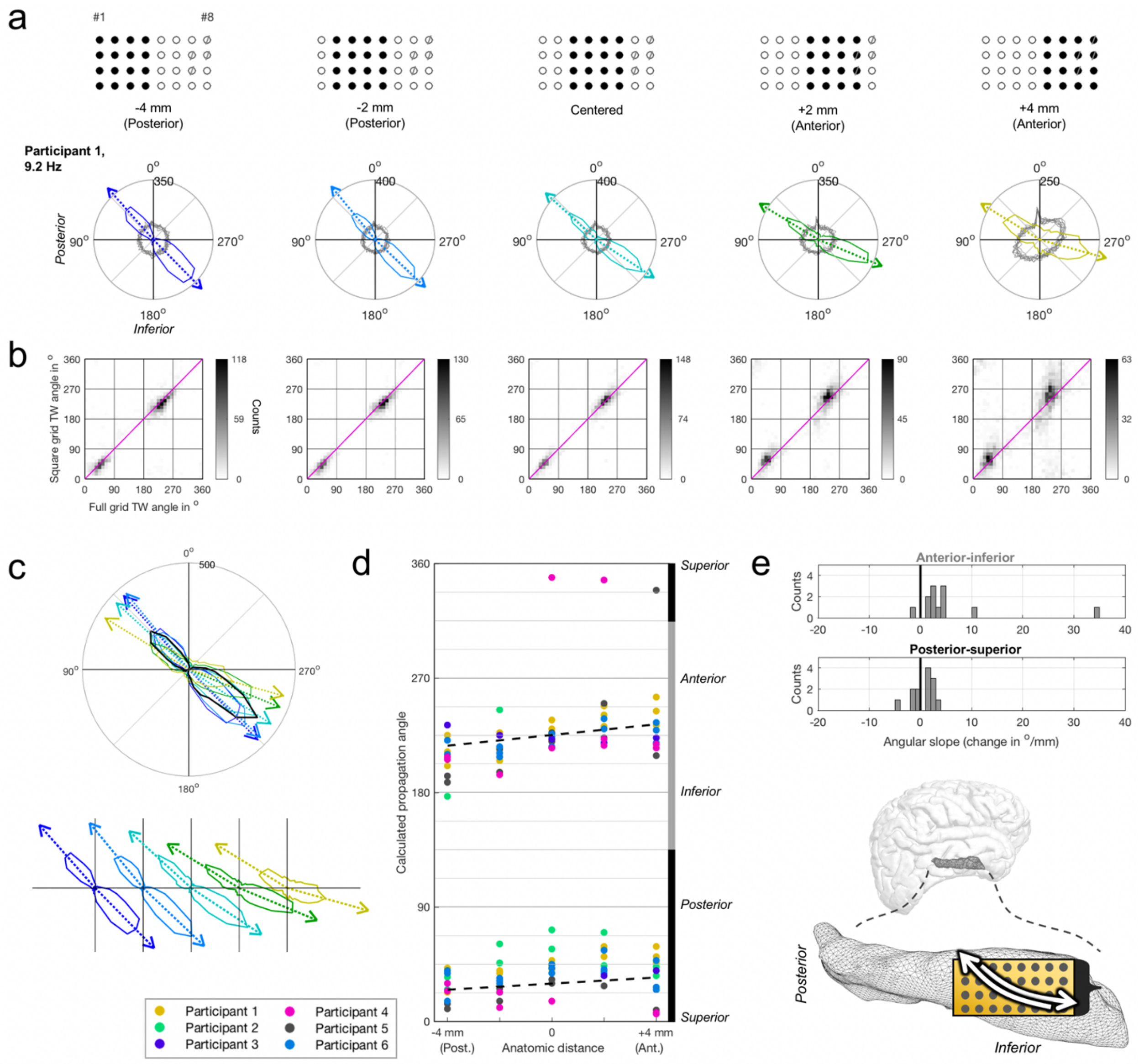

Supplementary Fig. 6.

Propagation angles along the hippocampal body. **a** Example propagation distributions as colored traces for (Participant 1, 9.2 Hz) when recalculating angles using subsampled square (4x4) configurations of electrodes advancing in 2mm steps from posterior to anterior (using the same timepoints as with full 4x8 grid in Supplementary Figs. 2 and 3; arrows: bimodal propagation angle estimates; grey traces: 10 example chance distributions from shuffling electrode coordinates). **b** Two-dimensional histograms comparing calculated traveling wave (TW) angle counts when using the full grid versus each of the subsampled square grids in a. Note that squares overlapping with electrodes excluded from the models in this participant (grey hatches, due to low/bad signal) created skewed electrode configurations, adding additional noise apparent in distributions in a and b. **c** Superimposing distributions (colored contours; original distribution from full grid in black for comparison) and modal/bimodal angles from a suggests a steady shift counterclockwise as one samples more anteriorly (also see subtle shifts from purple diagonal line in b). **d** Composite of all such modal directions across all participants (converting both R- and L-sided to a common anatomic axis). Trend lines are the median slope

values calculated separately for the anterior-inferior semicircle (grey sidebar) and separately for the posterior-superior semicircle (black sidebar) across all frequencies in all participants. **e** Histograms of angular slope values calculated with anterior-inferior and semicircles across all frequencies in all participants, with the majority being positive. Propagation appears weighted more along an inferior-superior axis closer to the septal pole and an anterior-posterior axis closer to the temporal pole, suiting the hippocampal curvature and perhaps explaining the prominent oblique directions when modeling on the full grid, though this was not formally assessed due to statistical constraints (circular data, bimodal distributions, repeated measures) and relatively limited observations.

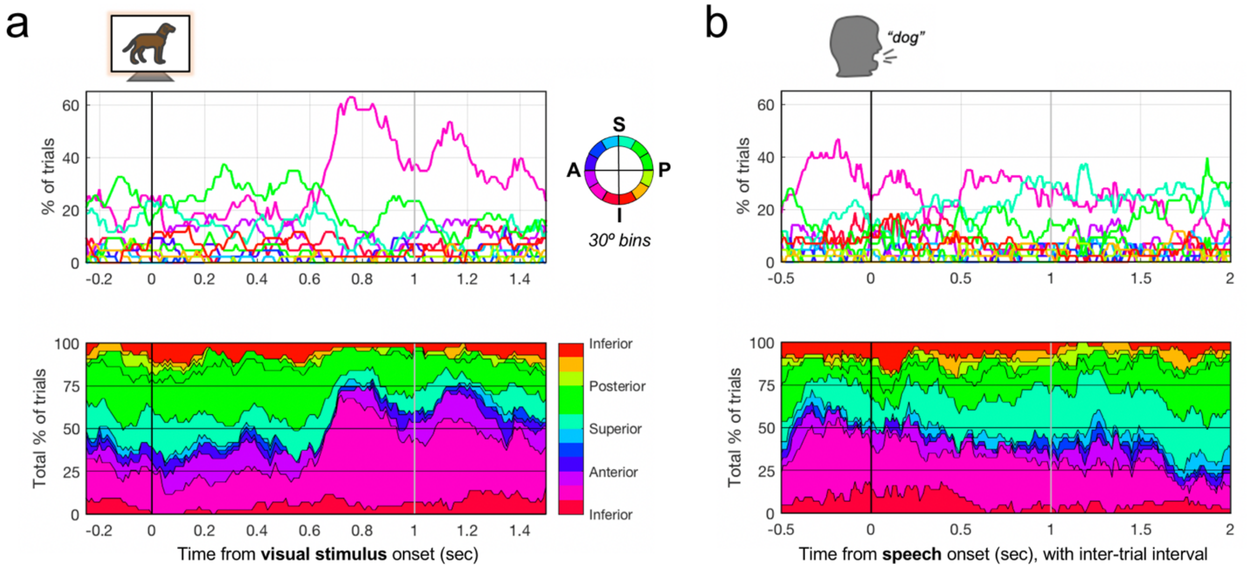

Supplementary Fig. 7.
Trial direction distributions. **a** Traces representing the proportions of trials angled toward each direction (upper panel; 30° bins, chance 8.33%; I: inferior, P: posterior, S: superior, A: anterior) at each timepoint, locked to the stimulus. Lower panel shows same data shown as a cumulative plot. See corresponding Fig. 3a. **b** Similar illustration but aligned to speech onset with the inter-trial interval shown (participant and data example from Fig. 4a). Propagation directions tended toward bimodal distributions when DC was low as expected by directional tendencies in the baseline period analysis (Fig. 2) but become more unimodal when DC was high.

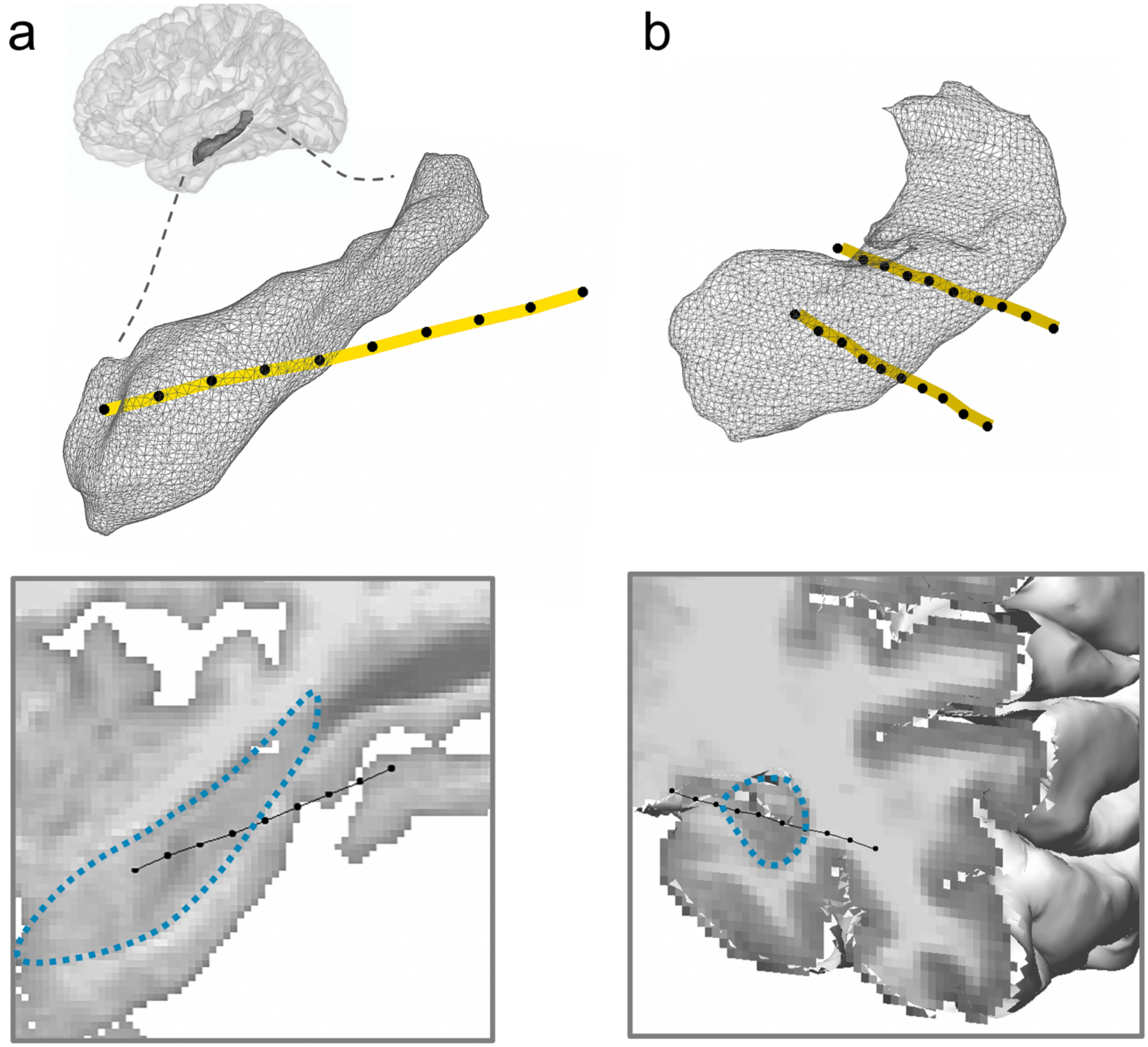

Supplementary Fig. 8.
Sampling limitations of typical human hippocampal depth recordings. **a** Digital reconstructions of a transparent left hemisphere and magnified human hippocampus (participant outside this study), illustrating an occipito-temporal approach with a 10-contact depth probe (yellow; contacts, black). A co-registered sagittal T1 MRI view shows its positioning (black) partially within the hippocampal body (blue outline) **b** Rotated view of hippocampal reconstruction using a lateral depth electrode approach (two depths, different participant also outside the study), along with a coronal T1 MRI view below (posterior depth shown; fused with temporal cortical surface and rotated to aid perspective). Both methods provide few electrode sites and have uneven and variable sampling of the hippocampal subfields.

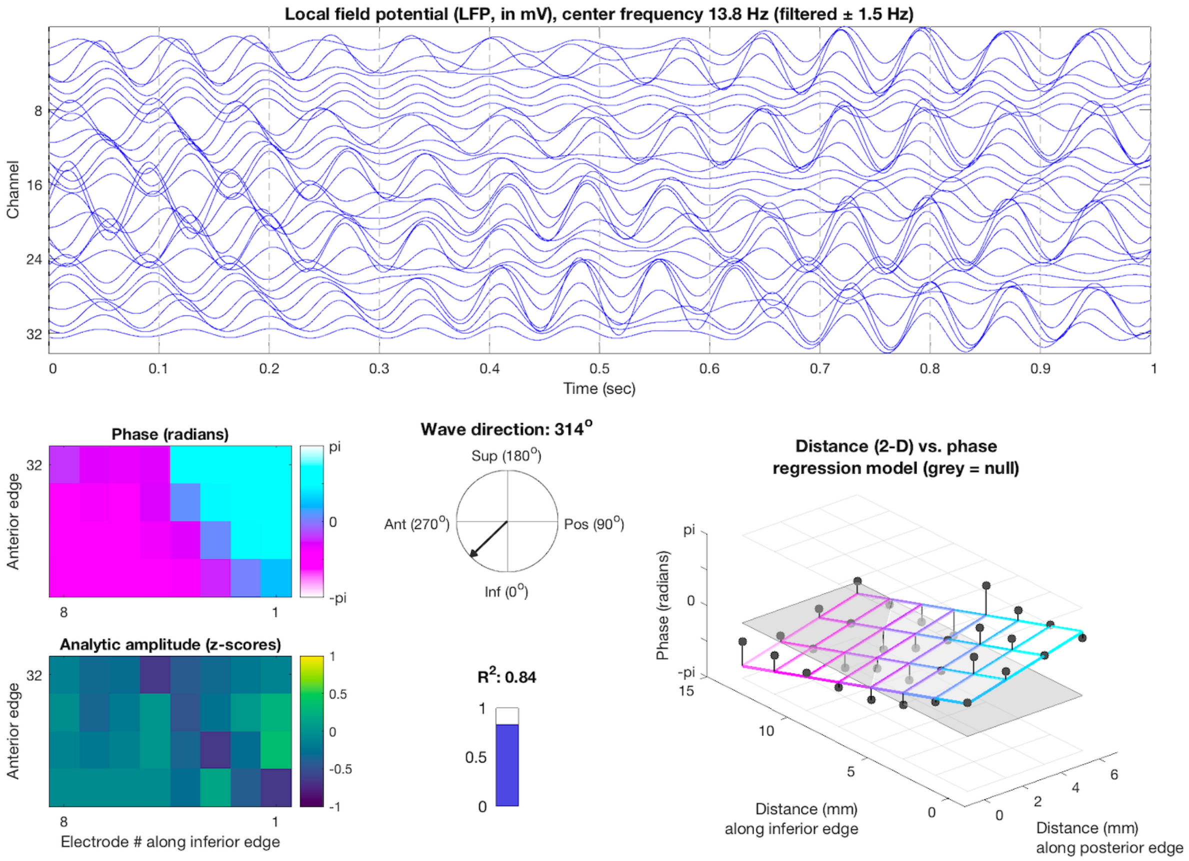

Supplementary Movie 1 [first frame shown above].

TW example and regression model. LFP waveform traces for a 1-second segment of the filtered 13.8 Hz oscillation example in Fig. 1 are shown at top. Vertical line marks timepoint of instantaneous analytics on bottom panels, including phase and amplitude in the bottom-left. Bottom right panel displays plane wave regression model (*x* and *y* axes: orthogonal anatomic dimensions, z-axis: phase). The plane model (colored grid) corresponds to the TW model-predicted phases (opacity: R^2), whereas actual phase values and residuals are the black dots and lines respectively. Calculated TW direction shown as an arrow (opacity: R^2) at center, with R^2 value as length of blue bar below. An antero-inferior traveling oscillation route is observed first, followed by a flat null model indicating poor overall directionality across the grid, and then a reemergence of the TW now coursing in a supero-posterior route. Frame rate is at 512 Hz sampling frequency (1.95 ms between frames).

Participant	Side	Condition	State	Behavior
1	Right	MTS	Anesthetized	N/A
2	Right	MTS	Anesthetized	N/A
3	Right	MTS	Anesthetized	N/A
4	Right	MTS	Anesthetized	N/A
5	Left	Tumor (Lateral Temporal Cortex)	Awake	Yes
6	Left	MTS	Awake	Yes*

Supplementary Table 1.
Participant characteristics. Six people (23-56 years old) participated in the study (four female). Four underwent right-sided surgery and hippocampal recording and two underwent the same surgery on the left (both awake for clinical stimulation mapping purposes). Both awake participants performed a behavioral (visual naming) task, though data from Participant 6 could not be analyzed due to interictal spike artifact among limited trials.* Most had mesial temporal sclerosis (MTS) as the clinical condition, though Participant 5 had a circumscribed left lateral temporal tumor (anterior middle temporal gyrus) and a presumably normal hippocampus.

Participant	Frequency (Hz)	-log(p-value)	Test	Angles (°)	Spatial Freq (°/mm)	Wavelength (mm)	Speed (mm/sec)
1	2.0	55	Hodges-Ajne	34	17.6	20.5	41.6
				214	16.4	21.9	44.5
	5.2	167	Hodges-Ajne	43	18.0	20.0	103.5
				222	14.8	24.3	126.1
	9.2	38	Hodges-Ajne	46	15.8	22.7	208.5
				235	13.0	27.6	253.1
2	4.4	87	Rayleigh	41	14.7	24.5	107.0
	6.9	88	Rayleigh	54	13.2	27.4	187.2
3	4.9	4	Hodges-Ajne	55	12.5	28.7	141.0
				230	12.0	29.9	146.8
4	1.7	16	Hodges-Ajne	17	15.6	23.1	39.2
				206	15.2	23.7	40.2
	4.6	86	Hodges-Ajne	7	11.9	30.1	138.1
				205	11.9	30.1	138.2
5	1.9	23	Hodges-Ajne	156	21.6	16.7	31.4
				323	17.1	21.1	39.6
	13.8	43	Hodges-Ajne	156	20.3	17.7	245.0
				323	18.3	19.7	272.4
6	1.6	1	Hodges-Ajne	149	15.8	22.8	36.2
				319	16.9	21.2	33.7
	9.9	35	Hodges-Ajne	128	14.6	24.7	245.0
				310	16.4	22.0	218.0
	12.4	37	Hodges-Ajne	139	16.3	22.1	273.5
				314	15.0	23.9	296.3

Supplementary Table 2.
Traveling oscillation characteristics. Individual detected peak frequencies and their results from the traveling oscillation analysis are shown. Regarding propagation direction (column 4, in degrees), note the circular orientation is flipped for right and left participants since the connector was anatomically oriented toward the temporal pole in all cases; refer to Fig. 2 and Supplementary Fig. 2 for anatomic orientation. Each oscillation was either unimodal or bimodal, and directionality significance was tested using Hodges-Ajne or Rayleigh tests respectively. Wave parameters averaged across all valid timepoints within 45° of each distinct angle are listed in righthand columns.

Participant	Freq. A (Hz)	Freq. B (Hz)	% ~Similar direction	% ~Opposite direction	R^2 rho (Spearman)	R^2 rho 99% C.I.	Speed rho (Spearman)	Speed rho 99% C.I.
1	2	5.2	57.6	42.4	0.061	-0.074 to 0.071	-0.047	-0.072 to 0.070
	2	9.2	51.4	48.6	0.044	-0.073 to 0.073	-0.035	-0.072 to 0.073
	5.2	9.2	56.5	43.5	0.047	-0.060 to 0.062	0.007	-0.061 to 0.062
2	4.4	6.9	69.5	30.5	0.022	-0.097 to 0.101	**0.107**	**-0.097 to 0.097**
3	4.9	N/A						
4	1.7	4.6	57.4	42.6	0.022	-0.117 to 0.120	0.108	-0.115 to 0.116
5	1.9	13.8	55.8	44.2	-0.023	-0.081 to 0.084	-0.005	-0.082 to 0.083
6	1.6	9.9	56.7	43.3	**-0.154**	**-0.148 to 0.149**	0.044	-0.154 to 0.150
	1.6	12.4	40.8	59.2	0.003	-0.150 to 0.153	0.107	-0.155 to 0.152
	9.9	12.4	65.7	34.3	-0.085	-0.110 to 0.112	**0.231**	**-0.117 to 0.111**

Supplementary Table 3.
Inter-frequency synchronization. For each participant with multiple peak frequencies, the table lists the proportion of timepoints in which a pair of frequencies traveled in a roughly similar direction (defined as Frequency A being within ± 90° (semicircle) angular distance of Frequency B, whereas an opposite direction was the other semicircle. Spearman rho values are also listed for inter-frequency correlations of R^2 (goodness of fit) and speed along with their 99% confidence intervals (10,000 iterations of shuffled timepoints). Significant correlations (bolded) were uncommon and not consistent across participants or frequencies.

Participant	Frequency (Hz)	Angles		Observations per angle	Classification Accuracy	99% CI
1	2	34	214	513, 1122	68.0%	44.4-55.1%
	5.2	43	222	583, 1827	75.4%	44.4-54.5%
	9.2	46	235	817, 1247	67.6%	45.5-53.9%
2	4.4	41		N/A		
	6.9	54		N/A		
3	4.9	55	230	498, 577	71.6%	44.3-55.1%
4	1.7	17	206	272, 244	89.3%	41.8-57.3%
	4.6	7	205	697, 338	91.6%	43.1-56.4%
5	1.9	156	323	655, 383	81.6%	43.6-55.6%
	13.8	156	323	1481, 711	70.3%	45-54.1%
6	1.6	149	319	161, 143	83.2%	39.5-59.1%
	9.9	128	310	158, 436	73.1%	40.2-58.9%
	12.4	139	314	147, 401	72.4%	39.5-59.5%

Supplementary Table 4.
Support vector machine model results. Individual detected peak frequencies and their bimodal directions (angle) are shown, along with the observations (total valid timepoints, at a 30 Hz resampled rate; see Methods) for each direction condition, the observed SVM model accuracy for each instance, and 99% confidence intervals (CI, for models in which direction labels were shuffled 10,000 times).
