# Supplementary material for: Bidirectional propagation of low frequency oscillations over the human hippocampal surface
Source: Nat Commun. 2021 May 12;12:2764. doi: 10.1038/s41467-021-22850-5 (PMC8115072; doi:10.1038/s41467-021-22850-5)
Supplement: Supplementary file 2 — Description of Additional Supplementary Files [file 41467_2021_22850_MOESM2_ESM.pdf]

Description of Additional Supplementary Files

File Name: Supplementary Movie 1
Description: TW example and regression model. LFP waveform traces for a 1- second segment of the filtered 13.8 Hz oscillation example in Fig. 1 are shown at top. Vertical line marks timepoint of instantaneous analytics on bottom panels, including phase and amplitude in the bottom-left. Bottom right panel displays plane wave regression model (x and y axes: orthogonal anatomic dimensions, z-axis: phase). The plane model (colored grid) corresponds to the TW model-predicted phases (opacity: R^2), whereas actual phase values and residuals are the black dots and lines respectively. Calculated TW direction shown as an arrow (opacity: R^2) at center, with R2 value as length of blue bar below. An antero-inferior traveling oscillation route is observed first, followed by a flat null model indicating poor overall directionality across the grid, and then a reemergence of the TW now coursing in a supero-posterior route. Frame rate is at 512 Hz sampling frequency (1.95 ms between frames).
