# Supplementary material for: Bidirectional propagation of low frequency oscillations over the human hippocampal surface
Source: Nat Commun. 2021 May 12;12:2764. doi: 10.1038/s41467-021-22850-5 (PMC8115072; doi:10.1038/s41467-021-22850-5)
Supplement: Supplementary file 4 — Reporting Summary [file 41467_2021_22850_MOESM4_ESM.pdf]

nature research

Corresponding author(s): Edward Chang

Last updated by author(s): 03/23/2021

Reporting Summary

Nature Research wishes to improve the reproducibility of the work that we publish. This form provides structure for consistency and transparency in reporting. For further information on Nature Research policies, see our Editorial Policies and the Editorial Policy Checklist.

Statistics

For all statistical analyses, confirm that the following items are present in the figure legend, table legend, main text, or Methods section.

n/a	Confirmed	
☐	☒	The exact sample size (*n*) for each experimental group/condition, given as a discrete number and unit of measurement
☐	☒	A statement on whether measurements were taken from distinct samples or whether the same sample was measured repeatedly
☐	☒	The statistical test(s) used AND whether they are one- or two-sided<br>*Only common tests should be described solely by name; describe more complex techniques in the Methods section.*
☐	☒	A description of all covariates tested
☐	☒	A description of any assumptions or corrections, such as tests of normality and adjustment for multiple comparisons
☐	☒	A full description of the statistical parameters including central tendency (e.g. means) or other basic estimates (e.g. regression coefficient) AND variation (e.g. standard deviation) or associated estimates of uncertainty (e.g. confidence intervals)
☐	☒	For null hypothesis testing, the test statistic (e.g. F, t, r) with confidence intervals, effect sizes, degrees of freedom and P value noted<br>*Give P values as exact values whenever suitable.*
☒	☐	For Bayesian analysis, information on the choice of priors and Markov chain Monte Carlo settings
☒	☐	For hierarchical and complex designs, identification of the appropriate level for tests and full reporting of outcomes
☐	☒	Estimates of effect sizes (e.g. Cohen's *d*, Pearson's *r*), indicating how they were calculated

Our web collection on statistics for biologists contains articles on many of the points above.

Software and code

Policy information about availability of computer code

Data collection	Data recordings were performed using TDT OpenEx software 2.31 from Tucker Davis Technologies. Microsoft Powerpoint version 2010 was used to deliver the visual stimuli.
Data analysis	Data processing and analyses were performed in Matlab R2019a, and all code is available from the corresponding author upon reasonable request. Custom automated-detection software for artifact (electrical, or interictal discharge) is publicly available from the Kleen Lab (https://github.com/Kleen-Lab), and circular-linear regression analysis was adapted from publicly available code from the Jacobs Lab (http://github.com/jacobslab/Traveling-wave-analysis). Hippocampal mesh reconstructions plotted and rotated in Matlab for illustrations were created from the publicly available resources Freesurfer (https://surfer.nmr.mgh.harvard.edu/) and the Chang Lab imaging pipeline (https://github.com/ChangLabUcsf/img_pipe).

For manuscripts utilizing custom algorithms or software that are central to the research but not yet described in published literature, software must be made available to editors and reviewers. We strongly encourage code deposition in a community repository (e.g. GitHub). See the Nature Research guidelines for submitting code & software for further information.

Data

Policy information about availability of data

All manuscripts must include a data availability statement. This statement should provide the following information, where applicable:

- Accession codes, unique identifiers, or web links for publicly available datasets
- A list of figures that have associated raw data
- A description of any restrictions on data availability

The de-identified data that support the findings of this study are available from the corresponding author on reasonable request. Source data for Figures 1-5 are provided with this paper and at DOI: 10.17605/osf.io/jbznx

April 2020

Field-specific reporting

Please select the one below that is the best fit for your research. If you are not sure, read the appropriate sections before making your selection.

[x] Life sciences [ ] Behavioural & social sciences [ ] Ecological, evolutionary & environmental sciences

For a reference copy of the document with all sections, see nature.com/documents/nr-reporting-summary-flat.pdf

Life sciences study design

All studies must disclose on these points even when the disclosure is negative.

Sample size	Sample-size calculations were not pre-determined since the amount of data collected from each participant was defined by intraoperative time constraints, and behavioral task data participation was additionally dependent on the amount of time each awake participant chose to volunteer.
Data exclusions	Data recording segments (or entire behavioral task trials) containing epileptiform discharges or electrical artifact were excluded from the analysis.
Replication	We did not explicitly attempt replicating the results described herein although traveling wave directional control analyses were performed independently on subsampled square grid for all six patients (Supp. Fig 3), several additional electrode constellations in two patients (Supp. Fig. 4), and a manual rotation control in one patient (Supp. Fig. 5), with all being successfully consistent with the original (rectangular grid) analysis.
Randomization	Randomization was not relevant for these experiments since the patient groups (awake vs. under anesthesia) were decided based solely on clinical necessity. For each iteration of SVM classification, models were trained on 80% of available data that was randomly selected (using the randperm.m function in Matlab) and the remaining 20% of data was held out until the testing phase of the finalized trained models.
Blinding	Blinding was not relevant for this study because it was decided clinically whether the participants were either awake or under anesthesia. If awake and participating in a behavioral task (visual naming) the experimenter did not interact with the participants aside from advancing the presented stimuli.

Reporting for specific materials, systems and methods

We require information from authors about some types of materials, experimental systems and methods used in many studies. Here, indicate whether each material, system or method listed is relevant to your study. If you are not sure if a list item applies to your research, read the appropriate section before selecting a response.

Materials & experimental systems

n/a	Involved in the study	
[x]	[ ]	Antibodies
[x]	[ ]	Eukaryotic cell lines
[x]	[ ]	Palaeontology and archaeology
[x]	[ ]	Animals and other organisms
[ ]	[x]	Human research participants
[x]	[ ]	Clinical data
[x]	[ ]	Dual use research of concern

Methods

n/a	Involved in the study	
[x]	[ ]	ChIP-seq
[x]	[ ]	Flow cytometry
[x]	[ ]	MRI-based neuroimaging

Human research participants

Policy information about studies involving human research participants

Population characteristics	Four participants were female and two were male, with ages between 23-56. Two participants were awake while undergoing a left-sided surgery, while the others were under anesthesia while undergoing a right-sided surgery. Five participants underwent this surgery for epilepsy and one underwent the surgery for a circumscribed left lateral temporal tumor.
Recruitment	We only recruited participants undergoing anterior temporal lobe surgery treatment for clinical reasons at the UCSF Medical Center, with an identical initial surgical approach thus avoiding bias from the type of operative procedure. Intraoperative monitoring was performed as part of routine clinical care, with the experimental use of high-density interelectrode spaced grids instead of standard clinical grid or strip spacing. All subjects underwent preoperative consent to participate in research and each participant was informed that any participation in this scientific research was completely voluntary and it would have no bearing on their clinical care. Participants were from male and female genders and of a wide age range, avoiding gender or age bias. Bias from most patients having epilepsy and associated hippocampal interictal discharges was controlled for by detection and exclusion of such discharges and the inclusion of a participant without epilepsy (circumscribed anterior temporal tumor with no imaging evidence of tumor invasion of the hippocampus; recordings confirmed no pathological epileptiform activity or loss of normal neurophysiological activity levels corroborating no tumor infiltration) who had a presumably normal hippocampus.
Ethics oversight	The experimental protocol was approved by the Institutional Review Board (Committee on Human Research) at the University of California San Francisco (UCSF).

Note that full information on the approval of the study protocol must also be provided in the manuscript.
